# The effect of food sensory experience on tourist engagement behavior: A study based on mental imagery theory

**DOI:** 10.1371/journal.pone.0351055

**Published:** 2026-06-11

**Authors:** Sen Yang, Yi Liu, Liping Xu

**Affiliations:** 1 School of Culture and Tourism, Hefei University, Hefei, People’s Republic of China; 2 Shenzhen Tourism College, Jinan University, Shenzhen, People’s Republic of China; 3 School of Marxism, Anhui Xinhua University, Hefei, People’s Republic of China; Sichuan Agricultural University, CHINA

## Abstract

Research on the effect of food tourism on tourist engagement behavior is an important gap in tourism research. Based on mental imagery theory, this study constructed a theoretical model to explore how food sensory experience influences tourist engagement behavior, with food memory (stored multisensory experiences of food) and mental imagery (active cognitive reconstruction of sensory information) as parallel mediating variables. This is the first study to integrate mental imagery theory into food tourism research, focusing on the core role of multisensory experience. A cross-sectional online survey was conducted targeting tourists who participated in Zibo’s food tourism (Shandong, China)—a typical case of post-epidemic tourism recovery driven by food—and 447 valid questionnaires were collected. Structural equation modeling (SEM) with bootstrapped indirect effects (sample size = 5000, 95% confidence interval) was used for data analysis. Results show that: (1) Food sensory experience (encompassing sight, smell, taste, and touch) exerts a significant positive effect on tourist engagement behavior, with the strongest impacts on feedback intention (β = 0.480, p < 0.001) and revisit intention (β = 0.411, p < 0.001); (2) Food memory and mental imagery play significant parallel mediating roles—sensory experience independently activates both constructs, which in turn drive engagement; (3) Mental imagery has a stronger mediating effect on recommendation intention than food memory, while food memory is more impactful on revisit intention. Theoretically, this study clarifies the sensory-cognitive-behavioral mechanism underlying tourist engagement, filling the gap of how multisensory experiences shape engagement behavior. Practically, it provides actionable strategies for tourism destinations to enhance tourist engagement. In addition, the implications and suggestions for future research are discussed and meaningful implications for relevant tourism destinations are provided.

## 1. Introduction

Food is becoming an increasingly important travel motivation [1] and a determining factor in tourists’ destination choice [2]. To cope with the difficulties and challenges posed by COVID-19 to the tourism industry, food has become an excellent means for destinations to attract tourists. Especially in the first half of 2023, the popularity of food tourism in Zibo, Shandong, China, played an important role in the recovery and development of tourism in the post-epidemic era. It has become a typical representative of the comprehensive recovery of tourism in China. Therefore, to quickly revive local tourism and gain competitive advantages, major tourist destinations have learned from Zibo to develop food tourism or local specialty tourism. More and more tourists are traveling to experience local food [3], as food, as one of everyone’s basic needs [4], increasingly drives tourist decisions.

Current research on the impacts and consequences of food tourism can generally be divided into macro and micro levels. At the macro level, food tourism is studied from the perspective of tourist destinations, mainly emphasizing the economic and cultural value of food tourism. Food tourism has become a key element in the development of tourist destinations and tourist experience, and an important tool for the protection, innovation and inheritance of local food heritage [5]. It is used as an important means to attract tourists and reshape the image of tourist destinations. Food image and brand value have become the core of tourism promotion [6], bringing high added value to tourist destinations and improving market competitiveness [7], as well as promoting the sustainable development of tourist destinations [8,9].

At the micro level, food tourism is studied from the perspective of tourists, emphasizing the experience value, experience intensification and extension of tourists. Studying the impact of food tourism experience on tourists’ attitudes and behaviors is the mainstream of food tourism research. Tourists’ food tourism experience is conducive to improving tourists’ satisfaction and enhancing tourists’ behavioral intentions [10]. Memory is an important outcome of food tourism experience [11], and food memory is more important than authenticity [12]. The experience of consuming local food can enable tourists to create positive and unforgettable memories, and this positive memory further enhances their sense of identification or strong attachment and behavioral intention to local attractions [3]. Scholars are increasingly paying attention to research on factors that influence tourists’ decision making, such as new technologies and electronic word-of-mouth, including digital presence and online interaction. A study on Instagram community found that residents were very supportive of attempts to promote native Portuguese cuisine through Instagram [13].

However, there are still three major research gaps in the study of food experience value, experience intensification and extension at the tourist level. First, it has not been clarified which specific experience has the most critical impact on tourist behavior. Research has shown that food tourism involves cognitive, emotional, behavioral, and sensory aspects [14]. Although some studies have categorized tourists’ food experience into multiple dimensions, [15], these dimensions are too detailed to capture the core experience that attracts tourists most. Food is the only product that evokes and engages all five human senses [14], the more sensory modalities are stimulated at any given time, the richer the customer experience will be [16,17], and the effect of sensory experience trumps emotional, behavioral, and intellectual experiences [18]. Therefore, studying the effect of sensory experience on tourists’ behavior has become an important research issue and a strategic demand for tourism destination construction.

Second, research on tourist engagement behavior in the context of food tourism has not received sufficient attention. The concept of tourist engagement stems from the broader field of customer engagement [19,20]. Marketing researchers debate whether customer engagement includes behavior [21]. Early studies viewed it as a psychological process [22], while recent research defines it as a set of behaviors [23]. Customer engagement behaviors can drive sales growth, recommendations, and competitive advantage [24], which has become a key indicator of customer relationship management [25]. Tourism is a highly engaging, enjoyable, emotional, and visual process that can trigger online discussions among tourists [26]. Moreover, tourists rely on the knowledge and creativity of other tourists in their travel experiences, which makes tourism environments unique in fostering tourist engagement [27]. However, research on tourist engagement has long been a neglected topic in the field of food tourism. Therefore, studying the impact of food tourism experiences on tourist engagement behaviors is an interesting topic that urgently needs to be addressed.

Third, there is a lack of empirical research on the influence mechanism of sensory experiences on tourist engagement behavior in the context of food tourism. Food is a unique form of tourism that inherently requires tourists to engage all their senses, thereby having a significant impact on their overall experience [18,28]. According to relevant studies, sensory experiences influence tourists’ memory and form memorable experiences [29], and these memorable experiences form tourists’ mental imagery and ultimately affect tourist revisit, recommendation, word-of-mouth, and feedback intention. However, there is a lack of explicit and systematic research on the mechanism of tourist food memory and mental imagery in this process.

To address the above gaps, this study aims to investigate the impact of food sensory experience on tourist engagement behavior (revisit, recommendation, word-of-mouth, feedback intentions) under the background of food tourism, and verify the parallel the mediating role of food memory and mental imagery. This study attempts to make three main contributions. First, it focuses on the core role of sensory experience in food tourism, overcoming the limitation of single-sense research in previous studies. Second, it is the first to introduce tourist engagement behavior into food tourism research, expanding the application scope of engagement behavior research. Third, it verifies the parallel mediating mechanism of food memory and mental imagery, clarifying the “sensory-cognitive-behavioral” link.

The remainder of this study is organized as follows. In part 2, the literature related to mental imagery theory, tourist engagement behavior, and the interaction of sensory experience, food memory, mental imagery, and tourist engagement behavior is reviewed, and research hypotheses are proposed. Then, part 3 describes the research method, and part 4 presents the analysis process and research results. Finally, part 5 discusses theoretical contributions and practical implications, followed by research limitations and future research directions.

## 2. Literature review and hypotheses development

### 2.1 Mental imagery theory

As an important theory in consumer psychology, mental imagery has received extensive attention in marketing and consumer behavior research. In marketing and consumer behavior research, the main focus is on mental imagery and live streaming effects [30], mental imagery and online product video demonstrations [31], mental imagery and consumer decision making [32], and mental imagery and purchase intention [33].

According to mental imagery theory, individuals mentally represent stimuli and actions based on past experiences and current perceptual information, automatically presenting previously experienced things or scenes in their minds [34]. Consumers can bring memorized sensory information into their minds through mental imagery evoked by external stimulus, which is considered a quasi-sensory experience [35]. Since mental imagery is a self-generated cognitive process [36], it can influence consumer attitudes and behaviors [37].

In the context of food tourism, mental imagery has distinct characteristics: (1) Modality-specific: It includes taste, smell, vision, and touch imagery, corresponding to the multisensory nature of food. (2) Vividness: The richness of sensory experience directly affects the vividness of mental imagery. (3) Motivational: Vivid food mental imagery can stimulate tourists’ behavioral intentions. From a consumer research perspective, consumer mental imagery of tourism products can become the main source of information to increase expectations and facilitate purchase decisions [38,39].

### 2.2 Tourist engagement behavior

Tourist engagement behavior originates from customer engagement behavior in the marketing field. Customer engagement behavior has become an important indicator for managing customer relationships [25], which can create value for enterprises [40,41]. Marketing researchers hold two perspectives on customer engagement [42], with the main distinction being the debate over whether it encompasses behavior [21]. Early on, customer engagement was conceptualized as a psychological process that drives consumer behavior [22,43] and positively influences customers’ positive reactions and behaviors [44].

Subsequent research has found that the various ways in which customers can contribute to a company are similar, and customer engagement is conceptualized as a set of different customer behaviors [23,45], such as providing reviews/feedback, spreading positive word-of-mouth, and/or recommending to others, all of which contribute simultaneously [46], and represent higher-order factors of long-term customer behavior [24]. In addition, Kumar et al. [47], Kumar and Pansari [23] believe that customer purchase is also a manifestation of customer engagement behavior. In this latest conceptualization, customer engagement is defined as the direct contribution (customer purchase) and/or indirect contribution (customer recommendation, influence (word-of-mouth), and feedback) made by customers to provide value to the firm [23,24].

It has also been initially studied in tourism consumer behavior, with the tourism environment having greater advantages for the formation of tourist engagement behavior [27]. Research has shown that hotel CSR [48], video marketing [49,50], and tourism social networking sites [51] can influence tourist engagement behavior. The ability of tourists to share their travel experiences online has provided a great impetus for the development of tourism social networking sites [52,53]. Therefore, tourism social networking sites have a greater attraction to tourists and a natural advantage in the formation of tourist engagement behaviors. Relevant tourism destinations and hotels can use tourism social networking sites to interact with tourists [54], receive tourist feedback [55], facilitate the formation of electronic word-of-mouth (eWOM) [56], and improve reputation and brand recognition [57]. Research has found that tourism social networking sites positively affect tourist engagement behavior [51,56], including transactional (customer purchases) and non-transactional (customer WOM, referrals, and feedback) [51]. In addition, some studies have found that the physical attractiveness of service employees [58] and service robots [59] also have a positive effect on tourist engagement behavior.

However, few studies on food tourism have focused on tourist engagement behavior. Currently, only relevant research on the impact of wine tourist motivations on tourist engagement in wine tourism has been found. Therefore, the study of tourist engagement behavior in food tourism is an interesting topic that has been neglected for a long time and has not received enough attention from relevant scholars, which deserves further consideration and research.

Therefore, this study draws on Kumar and Pansari [23] and Bravo et al. [51] to define tourist engagement behavior as a multidimensional construct, including: (1) Direct contribution: revisit intention to the destination; (2) Indirect contribution: recommendation intention, positive word-of-mouth, and feedback on services.

### 2.3 Food sensory experience and tourist engagement behavior

Sensory experience has been the focus of research in marketing and psychology, with relevant theories such as sensory marketing theory [60,61] and embodied cognition theory [62]. However, tourism scholars have paid less attention to this topic [29,63], and the role of the senses is a new research topic related to tourism [64]. The study of sensory experiences is crucial for tourism, and recent research has also highlighted the critical role of multisensory cues in the tourist experience [65]. Tourists interact with destinations through five senses, and food is the only tourism product that can arouse all senses [14], which has an impact on destination choice and future behavior [66]. Therefore, food sensory experience can be used as a tool to measure tourist experience and behavior.

Currently, there is no direct research on the effect of sensory experience on tourist engagement behavior, but there are relatively many studies on the effect of sensory experience on tourist revisit, recommendation, word-of-mouth, and feedback. Sensory experience is an important factor that influences customer purchase intention [14,67,68]. Sensory characteristics, such as taste, appearance and smell [69,70], play a very important role in motivating customers to purchase and consume [71], and sensory experience significantly affects customer food perception, consumption and purchase decisions [72]. A study on Korean street food demonstrated that the food experience quality has a positive impact on tourist word-of-mouth [73]. The sensory perception scale developed from five dimensions has verified that sensory perception is highly correlated with recommendation behavior and purchase behavior [74]. A study in hotel similarly proved that customer sensory experience has a significant impact on word-of-mouth and revisit [75]. According to a study on Chinese hotpot restaurant, customer experience has a positive impact on purchase, recommendation, word-of-mouth and feedback dimensions of customer engagement behavior [76]. Therefore, it is reasonable to believe that tourists with positive sensory experiences will revisit the destination and make suggestions to the destination to improve services, influence potential tourists through word-of-mouth and recommend to other tourists, such as through feedback, word-of-mouth and recommendation [23,45,47].

Therefore, this study proposes the following hypotheses:

**H1:** Food sensory experience has a positive effect on tourist engagement behavior.
**H1a:** Food sensory experience has a positive effect on tourist revisit intention.
**H1b:** Food sensory experience has a positive effect on tourist recommendation intention.
**H1c:** Food sensory experience has a positive effect on tourist word-of-mouth intention.
**H1d:** Food sensory experience has a positive effect on tourist feedback intention.

### 2.4 The mediating role of food memory

Memorable experiences refer to a person’s ability to easily recall events [77]. Providing memorable experiences is the new standard pursued by the tourism industry [75]. A large body of literature has proposed a link between food and memory [12,75,78], and memories of local food experiences has been identified as one of the most important outcomes of traveling to exotic places [12,79]. Tourists receive unique local food experiences that involve multiple senses, which are automatically associated with memories [80], and these food experiences are embedded in tourist memories [3,81]. Sensory experience is the key experience in memory formation [80,82]. Relevant research has confirmed that memory influences consumer future behavioral intentions [83], and tourist destination memories significantly influence tourist revisit intentions and recommendation intentions [3,83,84]. For example, tourists tend to revisit destinations that evoke positive memories [85]. Similarly, after tourists end their food tourism, the food sensory experience penetrates deep into their minds to form food memories [83], which further influences tourists’ word-of-mouth about the destination’s food and their feedback on related services.

Therefore, this study proposes the following hypotheses:

**H2:** Food memory mediates the effect of food sensory experience on tourist engagement behavior.
**H2a:** Food memory mediates the effect of food sensory experience on tourist revisit intention.
**H2b:** Food memory mediates the effect of food sensory experience on tourist recommendation intention.
**H2c:** Food memory mediates the effect of food sensory experience on tourist word-of-mouth intention.
**H2d:** Food memory mediates the effect of food sensory experience on tourist feedback intention.

### 2.5 The mediating role of mental imagery

Psychology and marketing have noted the importance of sensory perception on mental imagery [86,87]. However, few studies have investigated the relationship between food experiences and mental imagery in tourism. Sensory input is extremely important in creating mental imagery of a place [88], and consumers are able to visualize the sensory information in a situation even without direct sensory input later [89]. For example, the visual stimulation of online product displays can also promote mental imagery [86]. Visual perception of products is the driving force of mental imagery in retail shopping environments [87]. Mental imagery can be evoked by a variety of stimuli, including auditory, visual, tactile, gustatory, and olfactory stimuli [90]. Sensory perception occurs when information is recorded directly from the senses, while mental imagery occurs when an individual has a sensory experience by creating a mental image [87,91].

Conceptual distinction between food memory and mental imagery: Food memory is stored multisensory experiences of food (static, passive retention), while mental imagery is active cognitive reconstruction of sensory information (dynamic, active generation). The two constructs are parallel: sensory experience independently activates food memory and mental imagery, which then drive tourist engagement behavior (no sequential causal relationship).

Mental imagery-related research has been applied to a variety of consumer settings, and there is strong empirical support for the role of mental imagery in consumer behavior [86,87,92,93]. For example, digital menus are more likely to evoke vivid mental imagery than traditional menus, leading to greater enjoyment and higher behavioral intentions [93]. Visual stimulation of online products activates mental imagery, which further influences purchase intentions [86], and positive word-of-mouth [92]. Some studies consider providing feedback, helping others, and recommending services in customer engagement behaviors as extra-role customer engagement behaviors, which may not bring specific benefits to customers, but can benefit the company and contribute to the overall performance of the company [94,95]. Similarly, this study believes that tourists will also exhibit similar behaviors such as recommendation intention, word-of-mouth and feedback intention [96,97].

Therefore, this study proposes the following hypotheses:

**H3:** Mental imagery mediates the effect of food sensory experience on tourist engagement behavior.
**H3a:** Mental imagery mediates the effect of food sensory experience on tourist revisit intention.
**H3b:** Mental imagery mediates the effect of food sensory experience on tourist recommendation intention.
**H3c:** Mental imagery mediates the effect of food sensory experience on tourist word-of-mouth intention.
**H3d:** Mental imagery mediates the effect of food sensory experience on tourist feedback intention.

**Fig 1:**
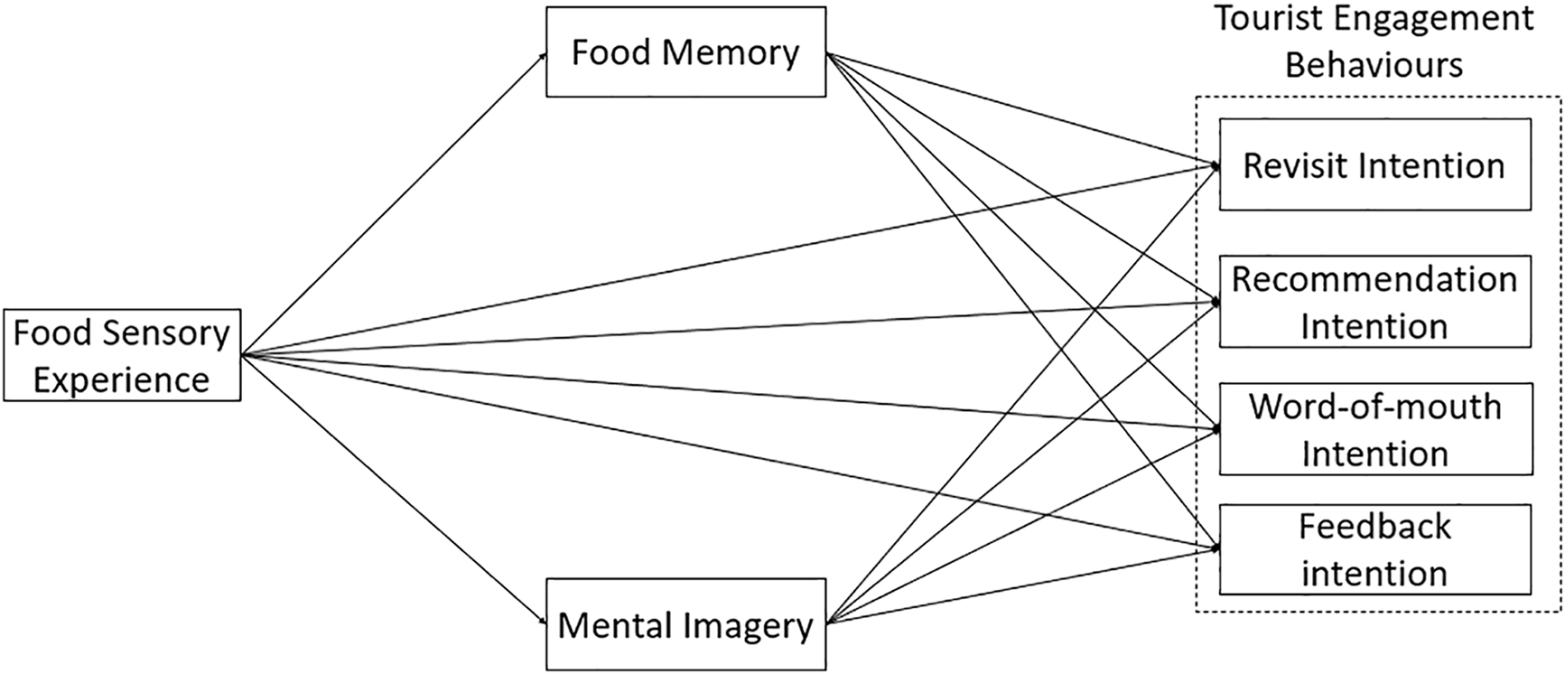
Theoretical model.

Based on the above literature review and research hypotheses, the theoretical model of this study was constructed as shown in Fig 1.

## 3. Research design

Ethics Statement: As our research does not directly involve the human body, our research has been exempted from ethics by the School of Culture and Tourism of Hefei University. All research methods are conducted in accordance with relevant guidelines and regulations, including those pertaining to data collection and analysis.

Informed Consent: Our research has obtained the informed consent of all participants. Before distributing questionnaires, we will ask tourists if they are willing to accept the survey; only after obtaining a positive answer do we send the questionnaire. We explain the purpose of the survey to respondents before starting the questionnaire. Minors were excluded because they lack independent travel decision-making capacity and complete food tourism experience, which is inconsistent with the research object definition. Tourists can withdraw consent at any link in the process, and withdrawn data are not included in the analysis. All participants received written informed consent.

### 3.1 Research background

In the first half of 2023, the barbecue in Zibo, Shandong, China became popular on the Internet, becoming a typical case of post-epidemic tourism recovery driven by food. Zibo, a national historical and cultural city, is undergoing industrial transformation and has made great efforts to create Zibo barbecue as a new city card.

The popularity of Zibo barbecue is mainly driven by tourists’ spontaneous sharing and feedback: tourists post food photos and videos on short video platforms to recommend to potential tourists, and provide timely feedback to merchants and destination management organizations (DMOs), forming a good reputation. Therefore, Zibo barbecue is an appropriate context to explore the influence of sensory experience on tourist engagement behavior.

### 3.2 Scale source and questionnaire design

The survey scale mainly consists of two parts. The first part includes scale items on food sensory experience, food memory, mental imagery and tourist engagement behavior, all of which refer to tourism literature and have good reliability and validity. The sensory experience items are mainly from Trinh et al. [98], with four items, sample items are “Zibo’s barbecue food smells nice” and “Zibo’s barbecue food looks nice”. The food memory items are mainly from Li, Su & Ma [2], with three items. The four items of mental image mainly refer to the scales of Huang, Wang & Chan [33] and Sun, Fang, Kong, Chen & Liu [99]. The tourist engagement behavior scale includes four dimensions of tourist revisit, recommendation, word-of-mouth, and feedback intention. Among them, the items for revisit intention and recommendation intention are mainly borrowed from Chen, Zhou, Zhan & Zhou [96], both of which are all three items. There are four items each for word-of-mouth intention and feedback intention, which are mainly adapted from the scale items of Yin, Li & Qiu [97]. The English scale was translated into Chinese and then back-translated into English by three tourism management Ph.D. students (one with overseas study experience) to ensure accuracy. Each item was measured using a five-point Likert scale (1 = strongly disagree, 5 = strongly agree). The second part is the demographic characteristic variables, mainly including gender, age, education, occupation, income, and tourist source. Scale specific information is presented in Table 1.

**Table 1:** Scale items and sources.

Latent Variables		Observed Variables	References
Sensory Experience		Zibo’s barbecue food smells nice	[98]
		Zibo’s barbecue food looks nice	
		Zibo’s barbecue food tastes good	
		Zibo’s barbecue food has a pleasant texture	
Food Memory		I had wonderful memories of Zibo’s food experiences	[2]
		I will remember many positive things about the food experiences of Zibo.	
		I will not forget my food experiences of Zibo.	
Mental Imagery		I can easily imagine eating the local food after food tourism.	[33,99]
		I can easily feel the flavors of local food after food tourism.	
		The image of food I have in my mind is intense.	
		The image of food I have in my mind is lifelike.	
Tourist Engagement Behaviors	Revisit Intention	If I could, I would come to Zibo again.	[96]
		I always consider Zibo as my first choice.	
		I have a strong intention to visit Zibo again.	
	Recommendation Intention	I will recommend others to visit Zibo in online forums.	
		I will talk about Zibo with others online.	
		My visits to Zibo are a natural topic of conversation online for me.	
	Word-of-mouth Intention	I do actively discuss Zibo on any media	[97]
		I love to talk about my food tourism experiences	
		I discuss the benefits that I get from Zibo with others.	
		I recommended Zibo and its services to others	
	Feedback Intention	I provide feedback about my experiences with the services to Zibo	
		I provide suggestions for improving the performance of the services.	
		I provide suggestions about the new product/services of Zibo	
		I provide suggestions for developing new products/services for Zibo	

### 3.3 Pre-survey

In May 2023, a pre-survey was conducted with 50 questionnaires collected to analyze the reliability and validity of the scale. The results show that the Cronbach’s coefficients of all variables are greater than 0.7, indicating that the scale has good internal consistency. In addition, the Kaiser-Meyer-Olkin (KMO) indices are all greater than 0.7, indicating good validity. Therefore, no changes were made to the final questionnaire.

### 3.4 Data collection

The formal questionnaire was collected from May to June 2023 through the Credamo questionnaire collection platform using convenience sampling. To ensure data quality, three polygraph questions were set (e.g., “Please select ‘strongly agree’ for this question”). In addition, pictures of Zibo barbecue were placed at the beginning of the questionnaire to stimulate tourist senses and evoke memories of their experiences. A total of 500 questionnaires were collected, of which 447 were valid, with an effective rate of 89.4%.

Although convenience sampling may limit generalizability, it is suitable for exploratory research on emerging phenomena (Zibo barbecue). Strict quality control measures (polygraph questions, validity screening) were adopted to ensure data reliability. The sample covers different genders, ages, and regions, which can provide preliminary insights into the research question.

## 4. Research results

### 4.1 Sample profile

Among the valid sample data collected, the majority are women (55.3%). The largest age group is 18–29 years old (36.9%). The highest level of education is junior college or bachelor’s degree (46.8%). The occupational distribution is relatively even, with slightly more private enterprise employees (22.7%). The maximum monthly income range is 6001–9000 (31.8%). Tourists mainly come from North China (40.9), which is the surrounding area where Zibo is located. The demographic details are shown in Table 2.

**Table 2:** Demographics (N = 447).

Variables	Categories	Frequency	Percentage of sample
Gender	Male	200	44.7
	Female	247	55.3
Age	18-29	165	36.9
	30-39	132	29.5
	40-49	66	14.8
	50-59	64	14.3
	>60	20	4.5
Education	High school and below	136	30.4
	Junior college or Bachelor’s degree	209	46.8
	Master’s degree	80	17.9
	Doctor’s degree	22	4.9
Occupation	Student	95	21.3
	Private enterprise employees	97	22.7
	Employees of state-owned enterprises	71	15.9
	Civil employed	69	15.4
	Freelancers	76	17.0
	Retirees	27	6.0
	Other	12	2.7
Monthly Income (Yuan)	≤3000	60	13.4
	3001–6000	112	25.1
	6001–9000	142	31.8
	9001–12,000	73	16.3
	12,001–15,000	41	9.2
	≥15,001	19	4.3
Geographic area	North China	183	40.9
	East China	72	16.1
	Northeast China	25	5.6
	Central China	51	11.4
	South China	81	18.1
	Northwest China	19	4.3
	Southwest China	16	3.6

### 4.2 Common method bias

Harman’s single factor test was used to test common method bias. All items of the scale that were not rotated were tested by principal component analysis, which showed that the KMO value was 0.905, with a total of 7 factors with eigenvalues greater than 1 extracted, and the cumulative variance explanation rate is 81.750%, of which the variance extracted by the first factor is 37.915%, which is less than the critical value of 40% [100]. In addition, the variance inflation factor for each regression was also examined, and the maximum value was 1.410, which is much lower than the critical value of 10. Therefore, this study concluded that there is no serious common method bias.

### 4.3 Reliability and validity analysis

In order to ensure the content validity, the study used a well-established questionnaire and referred to the comments of tourism Ph.D. students and tourists in tourism, which indicated that the content validity is good. Then, reliability analysis and confirmatory factor analysis were conducted using SPSS 27 and AMOS 28 software to test the reliability and convergent validity of the scale, and the results are shown in Table 3. Confirmatory factor analysis showed that the research model fit the actual data well: χ2/df = 2.288(1 < χ2/df < 5), GFI = 0.906(>0.9), AGFI = 0.878(>0.8), SRMR = 0.0427 (<0.1), RMR = 0.042(<0.05), RMSEA = 0.054(<0.08). NFI = 0.941(>0.9), RFI = 0.929(>0.9), IFI = 0.966(>0.9), TLI = 0.959(>0.9), CFI = 0.966(>0.9) [101]. The Cronbach’s α between variables ranged from 0.856 to 0.968, all of which are above the threshold of 0.7, indicating that the scale has good internal consistency. The factor loadings of each item are all greater than 0.5, with average variance extracted (AVE) ranging from 0.629–0.884, all exceeding the minimum value of 0.5, and composite reliabilities (CR) ranging from 0.835–0.968, all above 0.7 [102], indicating that the scale has good convergent validity.

**Table 3:** CFA results (N = 447).

Constructs	Items	Mean	Cronbach’s α	Standard factor loadings	t value	AVE	CR
Sensory Experience	SE1	3.85	0.901	0.772		0.690	0.899
	SE2	3.78		0.861	21.499		
	SE3	3.88		0.868	18.678		
	SE4	3.87		0.817	17.659		
Food Memory	FM1	3.38	0.856	0.845		0.670	0.859
	FM2	3.29		0.808	18.307		
	FM3	3.68		0.801	17.517		
Mental Imagery	MI1	2.89	0.968	0.896		0.884	0.968
	MI2	3.02		0.971	37.509		
	MI3	2.98		0.925	32.258		
	MI4	3.05		0.967	36.843		
Revisit Intention	REV1	3.89	0.863	0.726		0.629	0.835
	REV2	3.97		0.780	19.269		
	REV3	3.88		0.866	15.702		
Recommendation Intention	REC1	3.58	0.917	0.906		0.797	0.922
	REC2	3.74		0.952	32.457		
	REC3	4.06		0.815	23.587		
Word-of-mouth Intention	WOM1	4.06	0.897	0.801		0.699	0.901
	WOM2	4.04		0.824	18.116		
	WOM3	4.12		0.886	18.158		
	WOM4	4.21		0.830	18.190		
Feedback Intention	FB1	3.68	0.903	0.789		0.710	0.907
	FB2	3.89		0.891	21.507		
	FB3	3.94		0.895	21.288		
	FB4	4.04		0.789	17.891		

The correlation coefficients between the variables are presented in Table 4, and the diagonal line is the square root of the AVE value of the corresponding variable. As can be seen in Table 4, the maximum correlation coefficient is 0.717, and the minimum square roots of AVE is 0.793, and all correlation coefficients are smaller than the square root of AVE, indicating that the scale has good discriminant validity.

**Table 4:** Variable correlation coefficients and square root of AVEs (N = 447).

Constructs	Mean	S.D.	1	2	3	4	5	6	7
1.Sensory Experience	3.845	0.741	0.831						
2.Food Memory	3.448	1.051	0.451***	0.819					
3.Mental Imagery	2.986	1.222	0.401***	0.042	0.940				
4.Revisit Intention	3.912	0.707	0.688***	0.554***	0.417***	0.793			
5.Recommendation Intention	3.796	1.002	0.374***	0.254***	0.250***	0.304***	0.893		
6.Word-of-mouth Intention	4.110	0.767	0.363***	0.273***	0.217***	0.355***	0.538***	0.836	
7.Feedback Intention	3.887	0.671	0.669***	0.453***	0.404***	0.717***	0.365***	0.378***	0.843

Notes: ***p < 0.001.

### 4.4 Hypothesis testing

**Fig 2:**
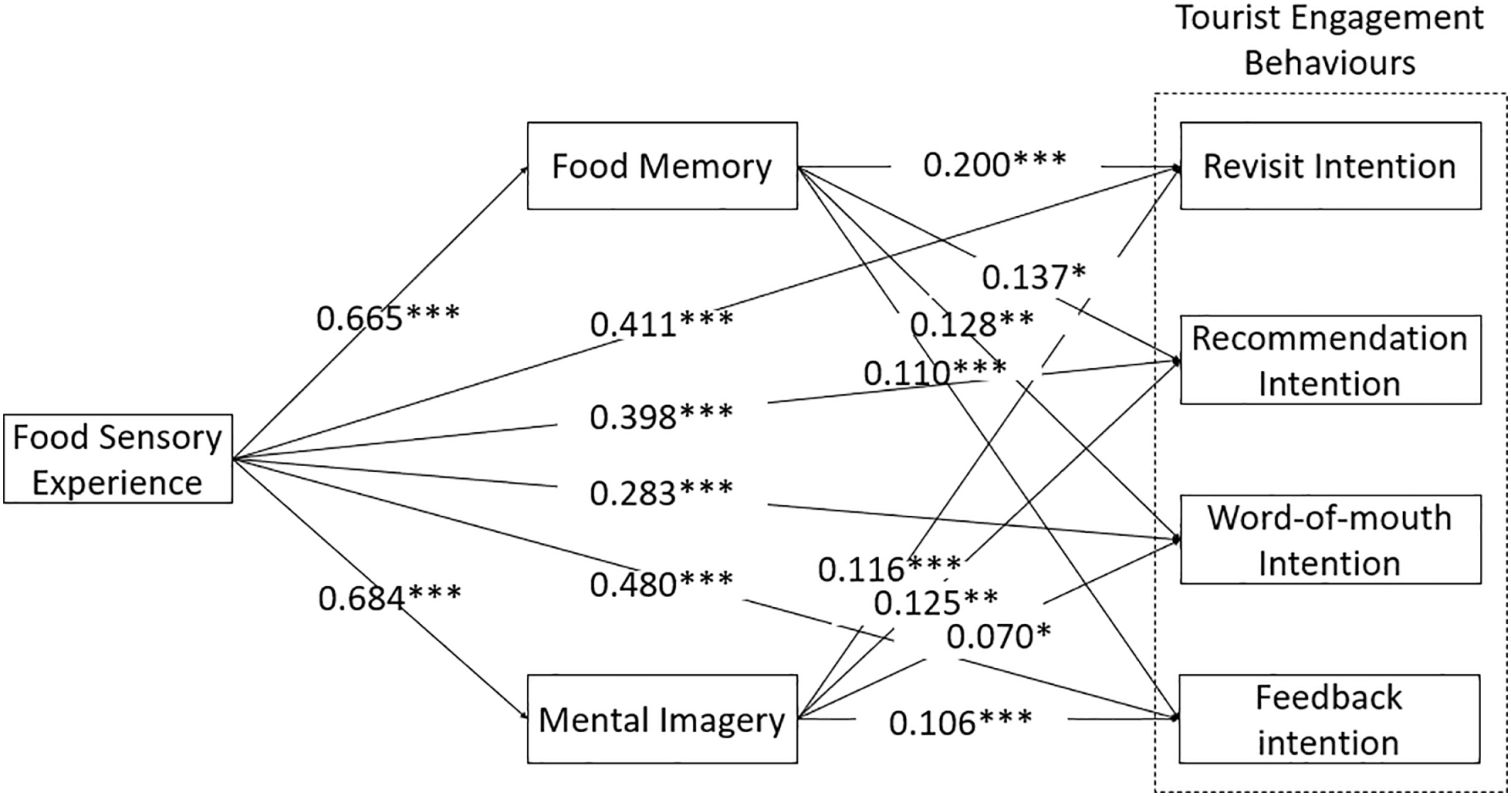
Structural equation model results. Notes: *p < 0.05, ** < 0.01, ***p < 0.001.

In this study, the maximum likelihood method was used to test the hypotheses of the structural model. The model fit reached an acceptable level: χ2 = 566.395, df = 253, p < 0.001, χ2/df = 2.239, GFI = 0.907, AGFI = 0.880, SRMR = 0.0492, RMR = 0.051, RMSEA = 0.053; NFI = 0.941, RFI = 0.931, IFI = 0.967, TLI = 0.960, CFI = 0.967 [101]. As can be seen from Fig 2 and Table 5, all hypotheses of H1 were verified, that is, food sensory experience has a significant positive effect on tourist engagement behavior. Specifically, food sensory experience has a positive and significant effect on tourist revisit intention (β = 0.411, p < 0.001), tourist recommendation intention (β = 0.398, p < 0.001), tourist word-of-mouth intention (β = 0.283, p < 0.001), and tourist feedback intention (β = 0.480, p < 0.001), which supports hypotheses H1a, H1b, H1c, and H1d.

**Table 5:** Structural model Results (hypothesis testing).

Hypotheses	Path	Standardized estimate	SE	C.R.	p	Results
H1a	SE → REV	0.411	0.054	7.646	***	Accepted
H1b	SE → REC	0.398	0.096	4.166	***	Accepted
H1c	SE → WOM	0.283	0.073	3.888	***	Accepted
H1d	SE → FB	0.480	0.055	8.679	***	Accepted

Notes: SE = food sensory experience, REV = tourist revisit intention, REC = tourist recommendation intention, WOM = tourist word-of-mouth intention, FB = tourist feedback intention, ***p < 0.001.

The strongest impact on feedback intention (β = 0.480) reflects that tourists are willing to provide feedback to optimize future experiences after obtaining high-quality sensory experiences. The strong impact on revisit intention (β = 0.411) indicates that sensory experience forms a strong emotional connection, prompting tourists to want to repeat the experience.

To test hypotheses H2 and H3, the bootstrap method was used to test the mediating effect of food memory and mental imagery between food sensory experience and tourist engagement behavior (PROCESS, model 4, sample size 5000, confidence interval 95%). As shown in Table 6, the confidence interval (CI) results of the bootstrapping method show that the CI of food memory in the mediating effect of food sensory experience on the four dimensions of tourist engagement behavior does not include zero (β_REV_ = 0.111, 95% CI = 0.067, 0.160; β_REC_ = 0.062, 95% CI = 0.012, 0.116; β_WOM_ = 0.055, 95% CI = 0.016, 0.096; β_FB_ = 0.087, 95% CI = 0.051, 0.128). Similarly, the CIs of mental imagery in the mediating effect of food sensory experience on the four dimensions of tourist engagement behavior do not include zero (β_REV_ = 0.075, 95% CI = 0.045, 0.106; β_REC_ = 0.086, 95% CI = 0.040, 0.138; β_WOM_ = 0.050, 95% CI = 0.015, 0.088; β_FB_ = 0.077, 95% CI = 0.047, 0.109). Therefore, the mediating effect of food memory and mental imagery is significant, supporting H2a, H2b, H2c, H2d, H3a, H3b, H3c, and H3d, that is, H2 and H3 are confirmed.

**Table 6:** Mediation test results.

Hypotheses	Path	Effect	SE	LLCI	ULCI	Results
H2a	SE → FM → REV	0.111	0.024	0.067	0.160	Accepted
H2b	SE → FM → REC	0.062	0.027	0.012	0.116	Accepted
H2c	SE → FM → WOM	0.055	0.020	0.016	0.096	Accepted
H2d	SE → FM → FB	0.087	0.020	0.051	0.128	Accepted
H3a	SE → MI → REV	0.075	0.016	0.045	0.106	Accepted
H3b	SE → MI → REC	0.086	0.025	0.040	0.138	Accepted
H3c	SE → MI → WOM	0.050	0.019	0.015	0.088	Accepted
H3d	SE → MI → FB	0.077	0.016	0.047	0.109	Accepted

Notes: SE = food sensory experience, FM = food memory, MI = mental imagery, REV = tourist revisit intention, REC = tourist recommendation intention, WOM = tourist word-of-mouth intention, FB = tourist feedback intention.

## 5. Conclusion and discussion

### 5.1 Conclusion

The role of food tourism in the development of tourist destinations has received widespread attention from destination management organizations and relevant scholars [2,3,5,6,8,9]. However, few studies have focused on the sensory experience of food tourists, and tourist engagement behavior in food tourism has not attracted the attention of relevant scholars. Therefore, based on mental imagery theory, this study explores the impact of food sensory experience on tourist engagement behavior and the mediating role of food memory and mental imagery. The main conclusions are as follows:

First, food sensory experience positively predicts tourist engagement behavior. Empirical results show that food sensory experience has a significant positive impact on tourist revisit, recommendation, word-of-mouth, and feedback intentions. This finding confirms that sensory experience influences tourist consumption and revisit intention [72,74,75], which has a positive impact on tourist recommendation behavior and word-of-mouth [73–75], and feedback the experience results [76]. Food sensory experience has a greater impact on tourist revisit and feedback intention than tourist recommendation and word-of-mouth intention. It may be because tourist sensory experience is enhanced while enjoying the food, and they are more likely to want to experience the food again and give feedback on the experience process, hoping to have a more perfect experience when they revisit next time.

Second, tourist food memory mediates the effect of food sensory experience on tourist engagement behavior. Specifically, food sensory experience forms food memories, which further affect tourist engagement behavior. Consistent with previous research, food sensory experience is the basis for food memory formation [82,80] and is deeply embedded in memory [3,81], which further affects tourist behavioral intentions, such as tourist revisit and recommendation intentions [3,83,84]. This confirms that food memory is an important link between sensory experience and tourist behavior.

Finally, tourist mental imagery mediates the effects of tourist sensory experiences on tourist engagement behavior. This is the first study to introduce tourist mental imagery into food tourism research, and explores its mediating role in the effects of tourist sensory experiences on tourist engagement behavior. The important role of mental imagery has been verified in various consumption settings [86,87,92,93]. Therefore, this study is consistent with the findings of Fan, Wong & Lin [88] and Kim, Kim, Park & Yoo [87]. After tourists enjoy the food sensory experience during food tourism, they are exposed to food stimulation, which leads to tourist mental imagery. Some studies have pointed out that mental imagery can be generated even without direct stimulation [89], which can have an important impact on tourist purchase intention [86] and positive word-of-mouth intention [92].

### 5.2 Theoretical enlightenment

Food tourism is an interesting and worthwhile research topic that has gained attention. The strong correlation between food and tourism and the contribution of food to tourism have been confirmed by most studies [81,103]. However, there are still several issues related to food tourism that have not yet been explored, that is, the research questions addressed and the theoretical contributions of this study.

First, although food experience is considered an important factor influencing tourist behavior, tourist food experience is rich and diverse [15], and sensory experience is at the core of food tourism experience, which requires the use of all senses to perceive and experience food [14]. This study captures the key experiential variable that influences tourist food behavior, which is sensory experience [18,28]. Based on mental imagery theory, this is the first study to explain how food sensory experience affects tourist engagement behavior. Moreover, food experience has been recognized as an important factor influencing destination choice and consumption [81,104], while previous studies have mainly conducted research from a single sense [64]. This study overcomes this limitation by studying food tourism from a multisensory perspective and drawing meaningful conclusions.

More importantly, this study is the first to examine tourist engagement behavior in food tourism. Customer engagement behavior has become a key indicator for managing customer relationships [25]. Previous research on customer engagement behavior has paid less attention to tourists as a special customer group, while tourism is precisely a highly engaged, enjoyable, and emotional process. and visual processes, which can elicit a wide range of discussions among tourists [26]. This study explores tourist engagement behavior, which has long been neglected in food tourism, which not only broadens the horizon of food tourism research, but also expands the application scope of tourist engagement behavior research. At the same time, it not only responds to tourist engagement behavior as a higher-order factor of long-term customer behavior that promotes consumption growth, customer recommendation, and gains competitive advantage [24], which plays a critical role in the long-term development of tourist destinations, but also fills the gaps and shortcomings of existing research on food tourism and tourist engagement behavior [105].

Finally, this study verified the mediating role of food memory and mental imagery. Food sensory experience has a direct effect on tourist behavior, such as consumer purchase intention [72] and behavioral intention [2], which extends their research and extends tourist behavior to tourist engagement behavior. It was verified that food memory and mental imagery play a dual mediating role in the effect of food sensory experience on tourist engagement behavior. Consistent with Li et al. [2], food memory mediates the effect of food sensory experience on tourist behavior. What is inconsistent is that this study takes food memory and mental imagery as dual mediators, while Li et al. [2] take food memory and destination attachment as dual mediators, and the effect of food memory is stronger in this study, and Li et al. [2] came up with the result that the mediating effect of memory is weaker, and the influence of food sensory experience on tourist behavior is mainly achieved through destination attachment. The possible reason is that tourists have not experienced food for too long, and this study used pictures to stimulate, as individual memories may gradually fade with time [106]. This study identified the mechanism conditions between food sensory experiences and tourist engagement behaviors, and found that positive food sensory experiences create food memories and mental imagery, which may lead to tourist engagement behaviors. At the same time, this study expands the application of mental imagery theory in food tourism and enhances the understanding of tourist mental imagery in food tourism.

A comparison of contradictory findings. Compared with Li et al. [2], this study finds that food memory has a stronger mediating effect, while Li et al. [2] argue that destination attachment is more important. The possible reason is that this study focuses on food-specific experience, and sensory experience directly forms food memory, while Li et al. [2] focus on overall destination experience.

### 5.3 Practical enlightenment

Food as a trigger for destination choice [81] and is increasingly becoming a source of attraction for tourist destinations [107], which can have a pulling effect on tourists. Therefore, some valuable suggestions are offered for relevant tourist destinations to better attract food tourists to experience and generate engagement behaviors.

#### 5.3.1 Optimize food sensory experience.

Food destination management organizations should strive to provide rich and satisfying sensory experiences, with food taste and visual enjoyment as the primary factors, in order to attract potential food tourists. Food is considered to be the only tourism product that evokes and involves all senses, and food sensory experience, as an important prerequisite for food memory and mental imagery, is determined to be the core of food experience and the experience that attracts the most attention from tourists. Relevant destinations should offer a rich and complete variety of dishes with ingredients that are authentic, fresh, clean and hygienic, serving mainly traditional or local flavor food, and ensure the taste and freshness of food to achieve bright colors, fragrant smell and delicious taste food as much as possible. Local traditional or local flavor food can be photographed or filmed in videos, and local food characteristics and traditional food culture can be organically integrated into the food experience, used to enhance the sensory experience of food, so that food tourists to the sight, sound, smell, taste, and touch an all-round sensory enjoyment and cultural experience.

#### 5.3.2 Strengthen online word-of-mouth management.

Strengthen the branding of local food, attach importance to the creation of online word-of-mouth on food social media, tourist recommendations, feedback and suggestions, so as to enhance the food tourist engagement behavior. Tourism destination management organizations should pay attention to food marketing and online word-of-mouth effects, and encourage tourists to spread word-of-mouth, recommendations and feedback on review websites, communities and travelogue sharing websites, and attach importance to and make full use of tourist consumption habits, such as taking photos to show off, check-in Internet-famous site, and sharing food pictures to seek self-identification, and guide food tourists to make positive comments on services, as well as food taste and local culture characteristics, so as to obtain positive word-of-mouth from food tourists, enhance food tourists’ group identity and consumption preference, and stimulate potential tourists travel intention.

#### 5.3.3 Construct food memory and mental imagery.

Focus on the formation of tourist food memory, and explore the construction of food tourist mental imagery. In order to form tourist engagement behavior and increase tourist revisit, word-of-mouth, recommendation and feedback intention, food tourism destinations should create memorable food experiences for tourists, and enhance tourists’ ability to form mental imagery. In addition to paying attention to tourist food sensory experience, relevant food festivals and food competitions can also be held, such as holding relevant food festivals allows tourists to fully participate in the whole process of food preparation, making and tasting, and holding food competitions for tourists to participate, and taking food photos, sharing food tweets and other activities, so that strengthen tourist emotional connection with tourist destination and form place attachment, thereby enhancing tourist memory and food mental imagery in the tourist destination, and finally achieve the purpose of tourist engagement behavior.

### 5.4 Limitations and future study

Although the study has some important findings, it still some limitations. First, the sampling method has limitations Because convenience sampling and single-city context (Zibo) may limit generalizability, and the sample is dominated by females, 18–29 age group, and North China tourists, which may lead to overestimation Thus future research should adopt multi-stage sampling and multi-city data collection to verify the model. Second, the research method relies on self-reported measures, which may have social desirability bias, so future research can adopt mixed-methods combining questionnaires with interviews and behavioral data to improve research validity. Third, this study only explores the mediating role of food memory and mental imagery without considering moderating variables (e.g., tourist involvement, food neophobia) and other mediating variables (e.g., place attachment), so future research can introduce these variables to construct a more comprehensive theoretical model. Fourth, the scale of tourist engagement behavior is adapted from existing literature, so future research can conduct in-depth interviews with food tourists to develop a special scale for food tourist engagement behavior.

## References

[1] Su DN, Johnson LW, O’Mahony B. Analysis of push and pull factors in food travel motivation. Curr Issues Tour. 2018;23(5):572–86. doi: 10.1080/13683500.2018.1553152

[2] Li S, Su Q, Ma J. How do food authenticity and sensory appeal influence tourist experience? The moderating role of food involvement. J Tour Res. 2022;25(1):109–22. doi: 10.1002/jtr.2552

[3] Tsai CT. Memorable tourist experiences and place attachment when consuming local food. J Tour Res. 2016;18(6):536–48. doi: 10.1002/jtr.2070

[4] Okumus B, Koseoglu MA, Ma F. Food and gastronomy research in tourism and hospitality: a bibliometric analysis. Int J Hosp Manag 2018;73:64–74. doi: 10.1016/j.ijhm.2018.01.020

[5] Lin M-P, Marine-Roig E, Llonch-Molina N. Gastronomy as a sign of the identity and cultural heritage of tourist destinations: a bibliometric analysis 2001–2020. Sustainability. 2021;13(22):12531. doi: 10.3390/su132212531

[6] Herman JL. Tracing Terroir(s): the role of maps, guidebooks, and regional products in constructing the French gastronomic imaginary. Food Cult Soc. 2023;27(1):4–25. doi: 10.1080/15528014.2023.2221139

[7] Barzallo-Neira C, Pulido-Fernández JI. Identification of the main lines of research in gastronomic tourism: a review of the literature. Sustainability. 2023;15(7):5971. doi: 10.3390/su15075971

[8] Sujood AR, Irfan S, Hamid S. Emerging themes in food tourism: a systematic literature review and research agenda. Br Food J. 2023. doi: 10.1108/BFJ-11-2022-0939

[9] Estrada M, Moliner MÁ, Monferrer D, Vidal L. Sustainability and local food at tourist destinations: a study from the transformative perspective. J Sustain Tour. 2023;32(5):1008–26. doi: 10.1080/09669582.2023.2195594

[10] Ali F, Ryu K, Hussain K. Influence of experiences on memories, satisfaction and behavioral intentions: a study of creative tourism. J Travel Tour Mark. 2015;33(1):85–100. doi: 10.1080/10548408.2015.1038418

[11] Richards G. Evolving research perspectives on food and gastronomic experiences in tourism. IJCHM. 2021;33(3):1037–58. doi: 10.1108/ijchm-10-2020-1217

[12] Stone MJ, Soulard J, Migacz S, Wolf E. Elements of memorable food, drink, and culinary tourism experiences. J Travel Res. 2017;57(8):1121–32. doi: 10.1177/0047287517729758

[13] Yu C-E, Sun R. The role of Instagram in the UNESCO’s creative city of gastronomy: a case study of Macau. Tour Manag. 2019;75:257–68. doi: 10.1016/j.tourman.2019.05.011

[14] Mohamed MEA, Hewedi MM, Lehto X, Maayouf M. Egyptian food experience of international visitors: a multidimensional approach. IJCHM. 2020;32(8):2593–611. doi: 10.1108/ijchm-02-2020-0136

[15] Badu-Baiden F, Kim S, Xiao H, Kim J. Understanding tourists’ memorable local food experiences and their consequences: the moderating role of food destination, neophobia and previous tasting experience. IJCHM. 2022;34(4):1515–42. doi: 10.1108/ijchm-06-2021-0709

[16] Everett S. Beyond the visual gaze? The pursuit of an embodied experience through food tourism. Tour Stud. 2008;8(3):337–58. doi: 10.1177/1468797608100594

[17] Schifferstein HNJ. From salad to bowl: the role of sensory analysis in product experience research. Food Qual Prefer. 2010;21(8):1059–67. doi: 10.1016/j.foodqual.2010.07.007

[18] Barnes SJ, Mattsson J, Sørensen F. Destination brand experience and visitor behavior: testing a scale in the tourism context. Ann Tour Res. 2014;48:121–39. doi: 10.1016/j.annals.2014.06.002

[19] Rasoolimanesh SM, Khoo-Lattimore C, Md Noor S, Jaafar M, Konar R. Tourist engagement and loyalty: gender matters? Curr Issues Tour. 2020;24(6):871–85. doi: 10.1080/13683500.2020.1765321

[20] Paul I, Roy G. Tourist’s engagement in eco-tourism: a review and research agenda. J Hosp Tour Manag. 2023;54:316–28. doi: 10.1016/j.jhtm.2023.01.002

[21] Honora A, Chih W, Ortiz J. What drives customer engagement after a service failure? The moderating role of customer trust. Int J Consum Stud. 2023;47(5):1714–32. doi: 10.1111/ijcs.12939

[22] Hollebeek LD. Demystifying customer brand engagement: exploring the loyalty nexus. J Mark Manag. 2010;27(7–8):785–807. doi: 10.1080/0267257x.2010.500132

[23] Kumar V, Pansari A. Competitive advantage through engagement. J Mark Res. 2016;53(4):497–514. doi: 10.1509/jmr.15.0044

[24] Pansari A, Kumar V. Customer engagement: the construct, antecedents, and consequences. J Acad Mark Sci. 2016;45(3):294–311. doi: 10.1007/s11747-016-0485-6

[25] Hollebeek LD, Sharma TG, Pandey R, Sanyal P, Clark MK. Fifteen years of customer engagement research: a bibliometric and network analysis. JPBM. 2021;31(2):293–309. doi: 10.1108/jpbm-01-2021-3301

[26] Zhou M, Yu H. Exploring how tourist engagement affects destination loyalty: the intermediary role of value and satisfaction. Sustainability. 2022;14(3):1621. doi: 10.3390/su14031621

[27] So KKF, King C, Sparks B. Customer engagement with tourism brands: scale development and validation. J Hosp Tour Res. 2014;38(3):304–29. doi: 10.1177/1096348012451456

[28] Lv X, Wu A. The role of extraordinary sensory experiences in shaping destination brand love: an empirical study. J Travel Tour Mark. 2021;38(2):179–93. doi: 10.1080/10548408.2021.1889447

[29] Agapito D, Pinto P, Mendes J. Tourists’ memories, sensory impressions and loyalty: in loco and post-visit study in Southwest Portugal. Tour Manag. 2017;58:108–18. doi: 10.1016/j.tourman.2016.10.015

[30] Wang B, Xie F, Kandampully J, Wang J. Increase hedonic products purchase intention through livestreaming: the mediating effects of mental imagery quality and customer trust. J Retail Consum Serv. 2022;69:103109. doi: 10.1016/j.jretconser.2022.103109

[31] Cheng Z, Shao B, Zhang Y. Effect of product presentation videos on consumers’ purchase intention: the role of perceived diagnosticity, mental imagery, and product rating. Front Psychol. 2022;13:812579. doi: 10.3389/fpsyg.2022.812579 35250742 PMC8891234

[32] Kim J-H, Kim M, Yoo J, Park M. Consumer decision-making in a retail store: the role of mental imagery and gender difference. IJRDM. 2020;49(3):421–45. doi: 10.1108/ijrdm-10-2019-0353

[33] Huang J, Wang L, Chan EY. Larger = more attractive? Image size on food packages influences purchase likelihood. Psychol Mark. 2022;39(6):1257–66. doi: 10.1002/mar.21644

[34] Lee W, Gretzel U. Designing persuasive destination websites: a mental imagery processing perspective. Tour Manag. 2012;33(5):1270–80. doi: 10.1016/j.tourman.2011.10.012

[35] Rodríguez-Ardura I, Martínez-López FJ. Another look at ‘being there’ experiences in digital media: exploring connections of telepresence with mental imagery. Comput Hum Behav. 2014;30:508–18. doi: 10.1016/j.chb.2013.06.016

[36] Escalas JE. Self‐referencing and persuasion: narrative transportation versus analytical elaboration. J Consum Res. 2007;33(4):421–9. doi: 10.1086/510216

[37] Alyahya M, McLean G. Examining tourism consumers’ attitudes and the role of sensory information in virtual reality experiences of a tourist destination. J Travel Res. 2021;61(7):1666–81. doi: 10.1177/00472875211037745

[38] Walters G, Sparks B, Herington C. The effectiveness of print advertising stimuli in evoking elaborate consumption visions for potential travelers. J Travel Res. 2007;46(1):24–34. doi: 10.1177/0047287507302376

[39] Cardoso L, Dias F, de Araújo AF, Andrés Marques MI. A destination imagery processing model: Structural differences between dream and favourite destinations. Ann Tour Res. 2019;74:81–94. doi: 10.1016/j.annals.2018.11.001

[40] Itani OS, El Haddad R, Kalra A. Exploring the role of extrovert-introvert customers’ personality prototype as a driver of customer engagement: Does relationship duration matter? J Retail Consum Serv. 2020;53:101980. doi: 10.1016/j.jretconser.2019.101980

[41] Carlson J, Rahman MM, Taylor A, Voola R. Feel the VIBE: Examining value-in-the-brand-page-experience and its impact on satisfaction and customer engagement behaviours in mobile social media. J Retail Consum Serv. 2019;46:149–62. doi: 10.1016/j.jretconser.2017.10.002

[42] Obilo OO, Chefor E, Saleh A. Revisiting the consumer brand engagement concept. J Bus Res. 2021;126:634–43. doi: 10.1016/j.jbusres.2019.12.023

[43] Brodie RJ, Hollebeek LD, Jurić B, Ilić A. Customer engagement: conceptual domain, fundamental propositions, and implications for research. J Serv Res. 2011;14(3):252–71. doi: 10.1177/1094670511411703

[44] Khan I, Hollebeek LD, Fatma M, Islam JU, Rahman Z. Brand engagement and experience in online services. JSM. 2019;34(2):163–75. doi: 10.1108/jsm-03-2019-0106

[45] Kumar V, Rajan B, Gupta S, Pozza ID. Customer engagement in service. J Acad Mark Sci. 2017;47(1):138–60. doi: 10.1007/s11747-017-0565-2

[46] Bijmolt THA, Leeflang PSH, Block F, Eisenbeiss M, Hardie BGS, Lemmens A, et al. Analytics for customer engagement. J Serv Res. 2010;13(3):341–56. doi: 10.1177/1094670510375603

[47] Kumar V, Aksoy L, Donkers B, Venkatesan R, Wiesel T, Tillmanns S. Undervalued or overvalued customers: capturing total customer engagement value. J Serv Res. 2010;13(3):297–310. doi: 10.1177/1094670510375602

[48] Ahn J. Role of hope and compulsion for CSR activities in hotel customers’ engagement. Curr Issues Tour. 2020;24(14):1958–64. doi: 10.1080/13683500.2020.1806797

[49] Cheng Y, Wei W, Zhang L. Seeing destinations through vlogs: implications for leveraging customer engagement behavior to increase travel intention. IJCHM. 2020;32(10):3227–48. doi: 10.1108/ijchm-04-2020-0319

[50] Qu F, Wang N, Zhang X, Wang L. Exploring the effect of use contexts on user engagement toward tourism short video platforms. Front Psychol. 2022;13:1050214. doi: 10.3389/fpsyg.2022.1050214 36506973 PMC9729704

[51] Bravo R, Catalán S, Pina JM. Understanding how customers engage with social tourism websites. JHTT. 2021;12(1):141–54. doi: 10.1108/jhtt-02-2019-0040

[52] Zhang H, Ji P, Wang J, Chen X. A novel decision support model for satisfactory restaurants utilizing social information: a case study of TripAdvisor.com. Tour Manag. 2017;59:281–97. doi: 10.1016/j.tourman.2016.08.010

[53] Oliveira B, Casais B. The importance of user-generated photos in restaurant selection. JHTT. 2019;10(1):2–14. doi: 10.1108/jhtt-11-2017-0130

[54] Hu L, Olivieri M. Social media management in the traveller’s customer journey: an analysis of the hospitality sector. Curr Issues Tour. 2020;24(12):1768–79. doi: 10.1080/13683500.2020.1819969

[55] Lu W, Stepchenkova S. User-generated content as a research mode in tourism and hospitality applications: topics, methods, and software. J Hosp Mark Manag. 2014;24(2):119–54. doi: 10.1080/19368623.2014.907758

[56] Malhan M, Dewani PP. Customer engagement on social networking sites: an experimental analysis in the tourism and hospitality sector. Curr Issues Tour. 2022;26(12):1915–40. doi: 10.1080/13683500.2022.2071685

[57] Kim W-H, Chae B. Understanding the relationship among resources, social media use and hotel performance. IJCHM. 2018;30(9):2888–907. doi: 10.1108/ijchm-02-2017-0085

[58] Fang S, Zhang C, Li Y. Physical attractiveness of service employees and customer engagement in tourism industry. Ann Tour Res. 2020;80:102756. doi: 10.1016/j.annals.2019.102756

[59] Li D, Liu C, Xie L. How do consumers engage with proactive service robots? The roles of interaction orientation and corporate reputation. IJCHM. 2022;34(11):3962–81. doi: 10.1108/ijchm-10-2021-1284

[60] Krishna A. An integrative review of sensory marketing: engaging the senses to affect perception, judgment and behavior. J Consum Psychol. 2011;22(3):332–51. doi: 10.1016/j.jcps.2011.08.003

[61] Agarwal S. Customer sense: how the 5 senses influence buying behaviour. J Consum Mark. 2015;32(4):307–8. doi: 10.1108/JCM-05-2014-0973

[62] Lv X, Lu R, Xu S, Sun J, Yang Y. Exploring visual embodiment effect in dark tourism: the influence of visual darkness on dark experience. Tour Manag. 2022;89:104438. doi: 10.1016/j.tourman.2021.104438

[63] Lv X, Li C, McCabe S. Expanding theory of tourists’ destination loyalty: the role of sensory impressions. Tour Manag. 2020;77:104026. doi: 10.1016/j.tourman.2019.104026

[64] Agapito D. The senses in tourism design: a bibliometric review. Ann Tour Res. 2020;83:102934. doi: 10.1016/j.annals.2020.102934

[65] Brochado A, Stoleriu O, Lupu C. Wine tourism: a multisensory experience. Curr Issues Tour. 2019;24(5):597–615. doi: 10.1080/13683500.2019.1649373

[66] Rousta A, Jamshidi D. Food tourism value: investigating the factors that influence tourists to revisit. J Vacat Mark. 2019;26(1):73–95. doi: 10.1177/1356766719858649

[67] Pramudya RC, Seo H-S. Hand-feel touch cues and their influences on consumer perception and behavior with respect to food products: a review. Foods. 2019;8(7):259. doi: 10.3390/foods8070259 31311188 PMC6678767

[68] Gracia A, Cantín CM. Effects of consumers’ sensory attributes perception on their willingness to pay for apple cultivars grown at different altitudes: are they different? Foods. 2022;11(19):3022. doi: 10.3390/foods11193022 36230098 PMC9562174

[69] Pula K, Parks CD, Ross CF. Regulatory focus and food choice motives. Prevention orientation associated with mood, convenience, and familiarity. Appetite. 2014;78:15–22. doi: 10.1016/j.appet.2014.02.015 24583413

[70] Tan HSG, Tibboel CJ, Stieger M. Why do unusual novel foods like insects lack sensory appeal? Investigating the underlying sensory perceptions. Food Qual Prefer. 2017;60:48–58. doi: 10.1016/j.foodqual.2017.03.012

[71] Roose G, Mulier L. Healthy advertising coming to its senses: the effectiveness of sensory appeals in healthy food advertising. Foods. 2020;9(1):51. doi: 10.3390/foods9010051 31948032 PMC7022983

[72] Imtiyaz H, Soni P, Yukongdi V. Role of sensory appeal, nutritional quality, safety, and health determinants on convenience food choice in an academic environment. Foods. 2021;10(2):345. doi: 10.3390/foods10020345 33562836 PMC7916031

[73] Lee S, Park H, Ahn Y. The influence of tourists’ experience of quality of street foods on destination’s image, life satisfaction, and word of mouth: the moderating impact of food neophobia. Int J Environ Res Public Health. 2019;17(1):163. doi: 10.3390/ijerph17010163 31881676 PMC6981621

[74] Haase J, Wiedmann K. The sensory perception item set (SPI): an exploratory effort to develop a holistic scale for sensory marketing. Psychol Mark. 2018;35(10):727–39. doi: 10.1002/mar.21130

[75] Liu C-R, Wang Y-C, Kuo TM, Chen H, Tsui C-H. Memorable dining experiences with five senses: conceptualization and scale development. J Hosp Tour Manag. 2022;53:198–207. doi: 10.1016/j.jhtm.2022.11.001

[76] Hussain K, Jing F, Junaid M, Zaman QU, Shi H. The role of co-creation experience in engaging customers with service brands. JPBM. 2020;30(1):12–27. doi: 10.1108/jpbm-08-2019-2537

[77] Kim J-H, Ritchie JRB, McCormick B. Development of a scale to measure memorable tourism experiences. J Travel Res. 2010;51(1):12–25. doi: 10.1177/0047287510385467

[78] Stone MJ, Migacz S, Sthapit E. Connections between culinary tourism experiences and memory. J Hosp Tour Res. 2021;46(4):797–807. doi: 10.1177/1096348021994171

[79] Williams HA, Yuan J, Williams RL Jr. Attributes of memorable gastro-tourists’ experiences. J Hosp Tour Res. 2018;43(3):327–48. doi: 10.1177/1096348018804621

[80] Badu‐Baiden F, Kim S. Is local food consumption memorable? Exploration of a multidimensional measurement scale to explain tourists’ memorable local food consumption experiences. Int J Tour Res. 2022;24(6):739–58. doi: 10.1002/jtr.2536

[81] Björk P, Kauppinen-Räisänen H. Exploring the multi-dimensionality of travellers’ culinary-gastronomic experiences. Curr Issues Tour. 2014;19(12):1260–80. doi: 10.1080/13683500.2013.868412

[82] Kauppinen‐Räisänen H, Gummerus J, Lehtola K. Remembered eating experiences described by the self, place, food, context and time. Br Food J. 2013;115(5):666–85. doi: 10.1108/00070701311331571

[83] Sharma P, Nayak JK. Understanding memorable tourism experiences as the determinants of tourists’ behaviour. J Tour Res. 2019;21(4):504–18. doi: 10.1002/jtr.2278

[84] Adongo CA, Anuga SW, Dayour F. Will they tell others to taste? International tourists’ experience of Ghanaian cuisines. Tour Manag Perspect. 2015;15:57–64. doi: 10.1016/j.tmp.2015.03.009

[85] Marschall S. Tourism and memory. Ann Tour Res. 2012;39(4):2216–9. doi: 10.1016/j.annals.2012.07.001

[86] Kim M. Digital product presentation, information processing, need for cognition and behavioral intent in digital commerce. J Retail Consum Serv. 2019;50:362–70. doi: 10.1016/j.jretconser.2018.07.011

[87] Kim M, Kim J-H, Park M, Yoo J. The roles of sensory perceptions and mental imagery in consumer decision-making. J Retail Consum Serv. 2021;61:102517. doi: 10.1016/j.jretconser.2021.102517

[88] Fan Y, Wong IA, Lin ZCJ. How folk music induces destination image: a synthesis between sensory marketing and cognitive balance theory. Tour Manag Perspect. 2023;47:101123. doi: 10.1016/j.tmp.2023.101123

[89] Argyriou E. Consumer intentions to revisit online retailers: a mental imagery account. Psychol Mark. 2012;29(1):25–35. doi: 10.1002/mar.20405

[90] MacInnis DJ, Price LL. The role of imagery in information processing: review and extensions. J Consum Res. 1987;13(4):473. doi: 10.1086/209082

[91] Yim MY-C, Baek TH, Sauer PL. I see myself in service and product consumptions: measuring self-transformative consumption vision (SCV) evoked by static and rich media. J Interact Mark. 2018;44(1):122–39. doi: 10.1016/j.intmar.2018.07.001

[92] Heller J, Chylinski M, de Ruyter K, Mahr D, Keeling DI. Let me imagine that for you: transforming the retail frontline through augmenting customer mental imagery ability. J Retail. 2019;95(2):94–114. doi: 10.1016/j.jretai.2019.03.005

[93] Yim MY-C, Yoo CY. Are digital menus really better than traditional menus? The mediating role of consumption visions and menu enjoyment. J Interact Mark. 2020;50(1):65–80. doi: 10.1016/j.intmar.2020.01.001

[94] Torkzadeh S, Zolfagharian M, Yazdanparast A, Gremler DD. From customer readiness to customer retention: the mediating role of customer psychological and behavioral engagement. EJM. 2022;56(7):1799–829. doi: 10.1108/ejm-03-2021-0213

[95] Sharma P. Destination evangelism and engagement: investigation from social media-based travel community. Electron Commer Res Appl. 2023;57:101228. doi: 10.1016/j.elerap.2022.101228

[96] Chen R, Zhou Z, Zhan G, Zhou N. The impact of destination brand authenticity and destination brand self-congruence on tourist loyalty: the mediating role of destination brand engagement. J Destin Mark Manag. 2020;15:100402. doi: 10.1016/j.jdmm.2019.100402

[97] Yin D, Li M, Qiu H. Do customers exhibit engagement behaviors in AI environments? The role of psychological benefits and technology readiness. Tour Manag. 2023;97:104745. doi: 10.1016/j.tourman.2023.104745

[98] Trinh HT, Dao BTT, Huynh TTT, Nguyen MTT, Nguyen TM, Vuong VT, et al. Diet quality index and food choice motives in vietnam: the roles of sensory appeal, mood, convenience, and familiarity. Foods. 2023;12:2505. doi: 10.3390/foods12132505 37444243 PMC10341352

[99] Sun C, Fang Y, Kong M, Chen X, Liu Y. Influence of augmented reality product display on consumers’ product attitudes: a product uncertainty reduction perspective. J Retail Consum Serv. 2022;64:102828. doi: 10.1016/j.jretconser.2021.102828

[100] Podsakoff PM, MacKenzie SB, Lee J-Y, Podsakoff NP. Common method biases in behavioral research: a critical review of the literature and recommended remedies. J Appl Psychol. 2003;88(5):879–903. doi: 10.1037/0021-9010.88.5.879 14516251

[101] Arpaci I, Baloğlu M. The impact of cultural collectivism on knowledge sharing among information technology majoring undergraduates. Comput Hum Behav. 2016;56:65–71. doi: 10.1016/j.chb.2015.11.031

[102] Fornell C, Larcker DF. Evaluating structural equation models with unobservable variables and measurement error. J Mark Res. 1981;18(1):39. doi: 10.2307/3151312

[103] Stone MJ, Migacz S, Wolf E. Beyond the journey: the lasting impact of culinary tourism activities. Curr Issues Tour 2018;22(2):147–52. doi: 10.1080/13683500.2018.1427705

[104] Kim YG, Eves A. Construction and validation of a scale to measure tourist motivation to consume local food. Tour Manag. 2012;33(6):1458–67. doi: 10.1016/j.tourman.2012.01.015

[105] Cheung M, Leung WKS, Taheri B, Tse SY. Driving destination brand engagement: the role of traveler participation. J Tour Res. 2023;25(6):565–80. doi: 10.1002/jtr.2594

[106] Farmaki A. Memory and forgetfulness in tourism crisis research. Tour Manag. 2021;83:104210. doi: 10.1016/j.tourman.2020.104210 32904475 PMC7456263

[107] Björk P, Kauppinen-Räisänen H. Local food: a source for destination attraction. IJCHM. 2016;28(1):177–94. doi: 10.1108/ijchm-05-2014-0214

